# Identification of 6 Hub Proteins and Protein Risk Signature of Colorectal Cancer

**DOI:** 10.1155/2020/6135060

**Published:** 2020-12-08

**Authors:** Taohua Yue, Cheng Liu, Jing Zhu, Zhihao Huang, Shihao Guo, Yuyang Zhang, Hao Xu, Yucun Liu, Pengyuan Wang, Shanwen Chen

**Affiliations:** Division of General Surgery, Peking University First Hospital, Peking University, 8 Xi Shiku Street, Beijing 100034, China

## Abstract

**Background:**

Colorectal cancer (CRC) is the second most common cause of cancer death in the United States and the third most common cancer globally. The incidence of CRC tends to be younger, and we urgently need a reliable prognostic assessment strategy.

**Methods:**

Protein expression profile and clinical information of 390 CRC patients/samples were downloaded from the TCPA and TCGA database, respectively. The Kaplan-Meier, Cox regression, and Pearson correlation analysis were applied in this study.

**Results:**

Based on the TCPA and TCGA database, we screened 6 hub proteins and first constructed protein risk signature, all of which were significantly associated with CRC patients' overall survival (OS). The risk score was an independent prognostic factor and significantly related with the size of the tumor in situ (*T*). 6 hub proteins were differentially expressed in cancer and normal tissues and in different CRC stages, which were validated at the ONCOMINE database. Next, 40 coexpressed proteins of 6 hub proteins were extracted from the TCPA database. In the protein-protein interaction (PPI) network, HER1, HER2, and CTNNB1 were at the center. Function enrichment analysis illustrated that 46 proteins were mainly involved in the EGFR (HER1) tyrosine kinase inhibitor resistance pathway.

**Conclusion:**

Studies indicated that 6 hub proteins might be considered as new targets for CRC therapies, and the protein risk signature can be used to predict the OS of CRC patients.

## 1. Introduction

According to the 2020 Colorectal Cancer Statistics, colorectal cancer (CRC) is the second most common cancer death in the United States [1]. The latest report points out that the age of onset of CRC is getting younger, with the median age dropping from 72 years in 2001-2002 to 66 years in 2015-2016 [2]. Especially for young CRC patients, we need tailored clinical management strategies, recognize the patient's risk in early diagnosis, reduce the side effects of treatment in low-risk patients, and perform adjuvant chemoradiotherapy other than surgery in high-risk patients.

Since the completion of the Human Genome Project and the rise of microarray profiling and genome-wide sequencing, increasing studies have predicted the survival of cancer patients at the genetic level [3–5]. Based on the Cancer Genome Atlas (TCGA) and the Gene Expression Omnibus (GEO), hundreds of differentially expressed, metastasis-related genes and survival-related genes have been identified in CRC tissues and cell lines [6–8]. Compared with utilizing a single gene to predict survival, the risk signatures or models constructed by multiple genes can predict the overall survival (OS) and disease-free survival more accurately [9]. Unfortunately, protein risk signature has never been constructed to predict the prognosis of CRC patients.

The Cancer Proteome Atlas (TCPA) database provided the protein expression profile by integrating RPPA chip data from TCGA and several independent tumor research projects [10]. In our research, based on the Kaplan-Meier method and Cox regression analysis, 6 hub proteins were identified, and a protein risk signature was firstly constructed. The overall survival (OS) of the high-risk group was significantly shorter than that of the low-risk group. The receiver operating characteristic (ROC) curve and the area under the ROC curve (AUC) further confirmed the accuracy of the risk signature. Pearson correlation analysis between the signature and clinical parameters indicated that the protein risk score of T_3_4 was significantly higher than that of T_1_2. With the help of the UALCAN website, we further found that 6 hub proteins were differentially expressed in cancer and normal tissues and in different CRC stages, which were validated at the ONCOMINE database. In order to mine the molecular characteristics of 6 hub proteins, we performed protein-protein interaction (PPI) and enrichment analysis based on the 6 hub proteins and their coexpressed proteins. We found that these proteins were mainly enriched in EGFR (HER1) tyrosine kinase inhibitor resistance, regulation of DNA metabolic process, and pathways in cancers, all of which were directly associated with tumorigenesis.

In conclusion, protein risk signature, a novel prognostic assessment tool, had the potential to predict the outcomes of CRC patients in clinical practice, and 6 hub proteins were expected to become novel therapeutic targets in the future.

## 2. Materials and Methods

### 2.1. Ethics Statement

All data in this study were obtained from online public databases and did not involve any in vitro or in vivo experiments.

### 2.2. Data Mining

The protein expression profiles and the matching clinical information of colorectal cancer (CRC) patients were downloaded from the Cancer Proteome Atlas Portal (TCPA) (https://www.tcpaportal.org/tcpa/) [11] and the Cancer Genome Atlas website (TCGA) (https://portal.gdc.cancer.gov/) [12], respectively. The TCPA is a comprehensive resource for accessing, visualizing, and analyzing functional proteomics of patient tumor samples and cancer cell lines. The TCGA is currently the largest cancer genetic information database and molecularly characterized over 20,000 primary cancer and matched normal samples covering 33 cancer types [13]. All the data in this study is the latest data from the above official website sources.

### 2.3. Identification of Candidate Proteins

The TCPA database had converted the raw data into a recognizable format [14]. Candidate proteins associated with the overall survival (OS) were extracted based on both univariate Cox proportional hazard regression analysis and Kaplan-Meier [15]. Proteins with a *p* value below 0.05 were defined as significant. Proteins with HR < 1 were defined as candidate protective proteins, while proteins with HR > 1 were considered candidate risky proteins [16].

### 2.4. Construction of the Prognostic Risk Signature

Based on the step-wise multivariate Cox proportional hazard regression analysis, we obtained 6 hub proteins from 24 candidate risk proteins [17]. The prognostic risk signature was built by combining the expression values of 6 hub proteins weighted by their regression coefficients. The median risk score was set as a cutoff value and divided CRC patients into high- and low-risk groups [18]. *R* “survival” package (https://CRAN.R-project.org/package=survival) was used to assess the significance of the OS difference between high- and low-risk groups.

### 2.5. Performance Assessment

To measure the performance of our hub protein risk signature, the receiver operating characteristic (ROC) curve and the corresponding areas under the ROC curve (AUC) were produced using the *R* “survivalROC” packages [19]. The univariate and multivariate Cox proportional hazard regression analyses were performed to evaluate the independent prognostic potential of protein risk signature. Multivariate Cox analysis adjusted the influences of age, gender, pathological American Joint Committee on Cancer (AJCC) stage, tumor size in situ (*T*), lymph node metastasis (*N*), and distant metastasis status (*M*) on risk signature [20].

### 2.6. Clinical Parameter Correlation

Based on the TCGA clinical information, we performed the Pearson correlation analysis between risk signature and age, gender, stage, *T*, *N*, and *M* [21].

### 2.7. Differential Expression Analysis

UALCAN is a user-friendly and interactive web resource for analyzing cancer OMICS data based on level 3 RNA-seq data and clinical data of 31 cancer types from the TCGA database [22]. The differential expression of 6 hub proteins and their encoding genes were analyzed at the UALCAN website (http://ualcan.path.uab.edu/) [23].

The ONCOMINE database (http://www.oncomine.org/) is a cancer microarray database and integrated data-mining platform designed to facilitate discovery from genome-wide expression analysis [24]. In our research, transcriptional data of 6 encoding genes between CRC tissues and normal tissue were obtained from this database. Thresholds were designed as follows: *p* value: 0.001, fold change: 1.5, gene rank: top 10%, data type: mRNA.

### 2.8. Protein Coexpression Analysis

Based on 6 hub proteins, we performed Pearson correlation analysis and found their coexpressed proteins. The correlation filtering criteria were *p* less than 0.001, and Pearson correlation coefficient (PCC) greater than 0.4. PCC > 0 meant a positive correlation with hub protein, and PCC < 0 meant a negative correlation. Next, we generated a Sankey diagram using ggplot2 and ggalluvial package among 6 hub proteins and their coexpressed proteins.

### 2.9. Molecular Network and Functional Enrichment Analysis

Protein-protein interaction (PPI) and functional enrichment analysis were constructed using the STRING database (https://string-db.org/), Cytoscape software version 3.7.1 [25], and the Metascape (https://metascape.org/gp/index.html) [26]. The STRING database helps to explore the interaction network between proteins and discover core regulatory proteins. If the network contained between 3 and 500 proteins, the Molecular Complex Detection (MCODE) algorithm would be applied to identify densely connected network components [26]. To clearly illustrate the functions and molecular pathways of 6 hub proteins and their coexpressed proteins, we conducted the Gene Ontology (GO) enrichment analysis and Kyoto Encyclopedia of Genes and Genomes (KEGG) analysis at the Metascape website [27].

### 2.10. Statistical Analysis

In this study, all statistical analyses were performed using *R* software (version 3.6.1; https://www.r-project.org/). Significance was defined as *p* < 0.05.

## 3. Results

### 3.1. Identification of Candidate Survival-Related Proteins

**Figure 1:**
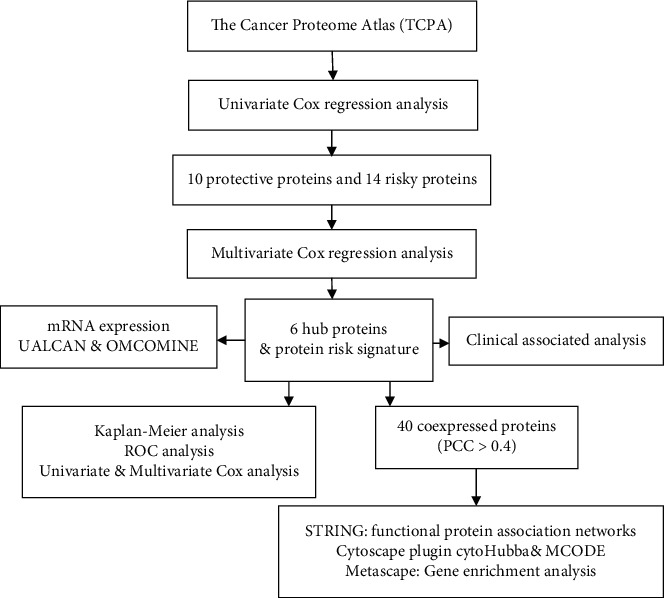
Flow chart of our study.

**Figure 2:**
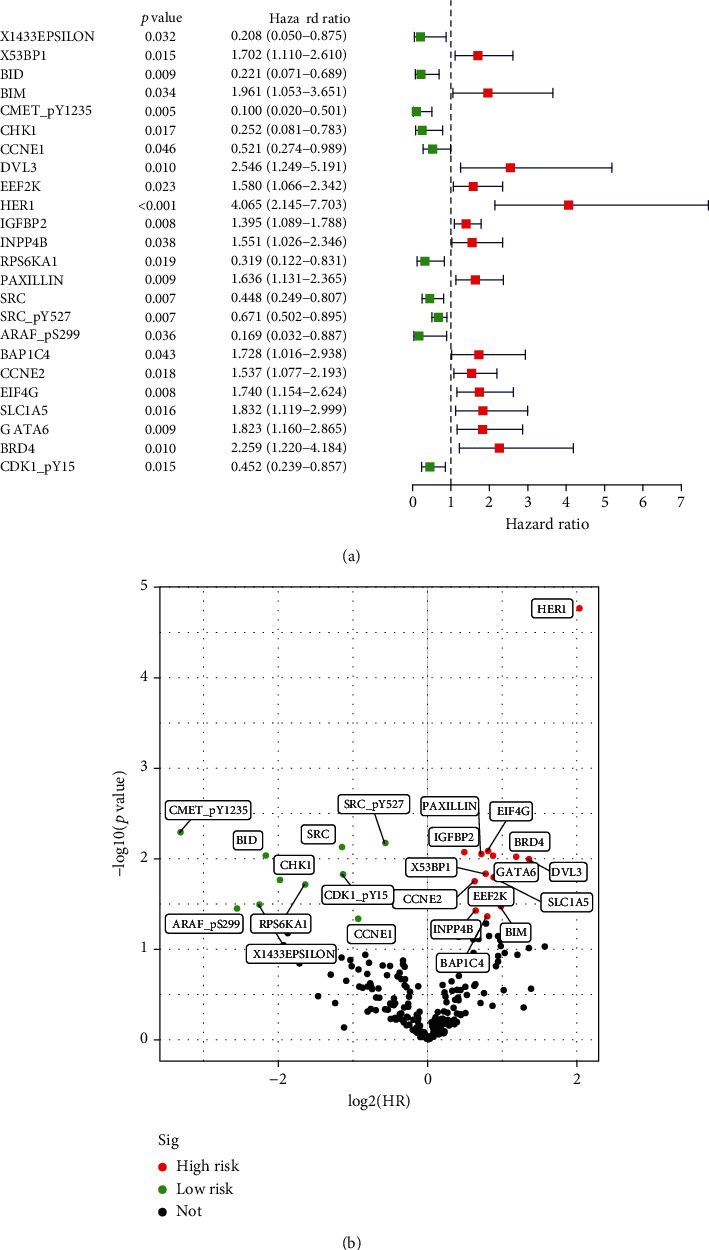
24 candidate proteins were significantly correlated to prognosis in CRC. (a) Forest plot of 24 candidate proteins. (b) Volcano plot of 24 candidate proteins. The green dots indicated low-risk proteins, while the red dots represented high-risk proteins. *p* < 0.05.

The workflow of our study was illustrated in Figure 1. At the TCPA database, we downloaded protein expression profile of 390 CRC patients/tissues. The corresponding clinical information was downloaded from the TCGA database. There was no requirement for ethical approvals. As predicting the prognosis of CRC that was critical for cancer patients, we performed univariate Cox regression analysis and explored 24 candidate survival-associated proteins, including 10 protective proteins and 14 risky proteins, all of which were displayed in the forest plot (Figure 2(a)) and volcano plot (Figure 2(b)). Risky proteins meant that the higher its expression, the higher the patient's risk of death and the shorter the overall survival (OS).

### 3.2. Identification of Hub Proteins

**Figure 3:**
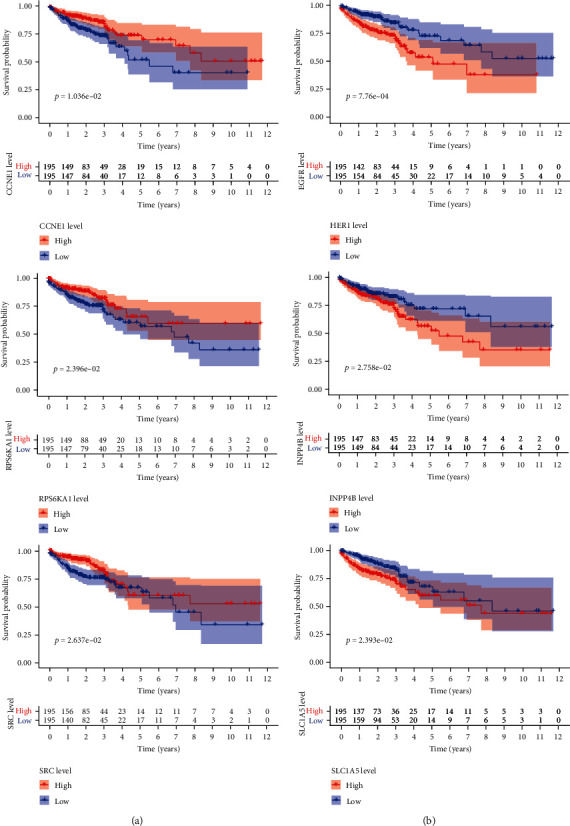
Kaplan-Meier survival curve among high- and low-risk groups based on 6 hub proteins. (a) The higher the expression of CCNE1, RPS6KA1, and SRC, the longer the OS of CRC patients. (b) The higher the expression of HER1, INPP4B, and SLC1A5, the shorter the OS of CRC patients.

To extract hub proteins that were actively involved in the onset and progression of CRC, we further performed multivariate Cox regression analysis and finally identified 6 survival-related proteins, CCNE1, HER1, INPP4B, RPS6KA1, SRC, and SLC1A5, and named them hub proteins (*p* < 0.05). All of the identified hub proteins were significantly related to the OS of CRC patients and were potential prognostic markers for monitoring patients' outcomes (Figure 3).

### 3.3. Construction and Validation of a Prognostic Signature

**Figure 4:**
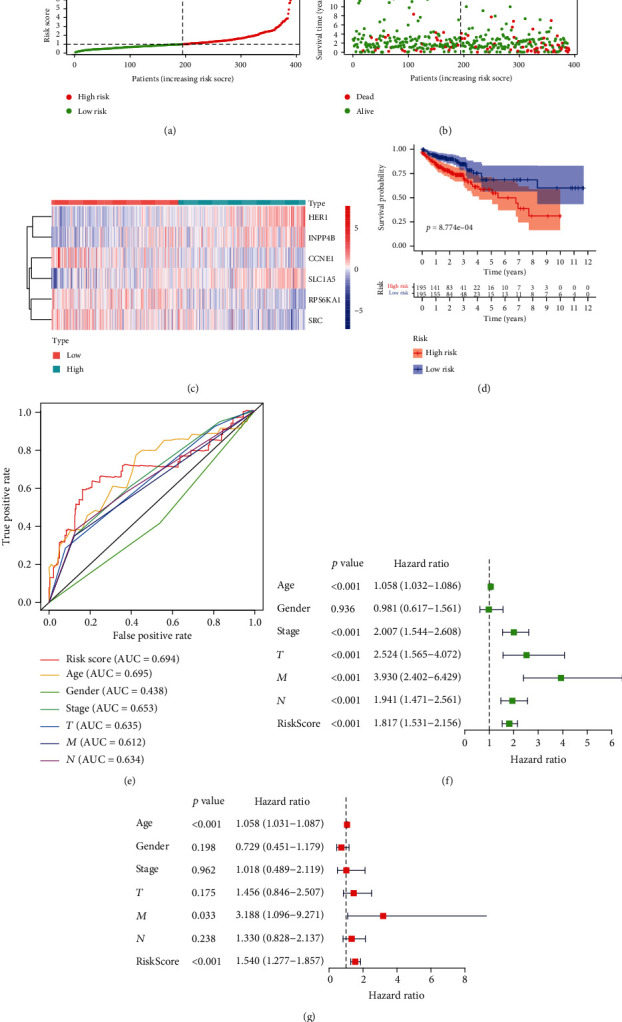
Construction and validation of protein risk signature. (a) Risk score distribution of CRC patients based on the median of risk score (low, green; high, red). (b) Scatterplots of CRC patients with different survival status in both groups. (c) Heatmap of expression profiles of included 6 hub proteins. (d) Kaplan-Meier curve of the high-risk (red) and low-risk (blue) CRC patients and patients in the high-risk group had a shorter overall survival. (e) The predictive accuracy of risk signature. (f) Univariate and (g) multivariate Cox regression analysis to verify that the risk score was an independent prognostic factor.

Based on multivariate Cox regression analysis, we further constructed a protein risk signature, and the formula was as follows: [expressions of CCNE1 × (−0.7475)] + [expressions of HER1 × 0.8318] + [expressions of INPP4B × 0.3337] + [expressions of RPS6KA1 × (−0.8763)] + [expressions of SRC × (−0.7350)] + [expressions of SLC1A5 × 0.6579]. Based on the median of the risk score, we separated CRC patients into 2 groups, the high- and low-risk group (Figure 4(a)). CRC patients belonging to the high-risk group doomed to a poor prognosis, and the number of patients who died of CRC was significantly more than that of the low-risk group (Figure 4(b)). The differential expression of 6 hub proteins between the high- and low-risk groups was illustrated in the heat map. HER1, INPP4B, and SLC1A5 were highly expressed in the high-risk group, while CCNE1, RPS6KA1, and SRC were highly expressed in the low-risk group (Figure 4(c)). The protein risk signature was significantly associated with the OS of CRC patients. The OS in the high-risk group was significantly shorter than that of the low-risk group (Figure 4(d)).

**Figure 5:**
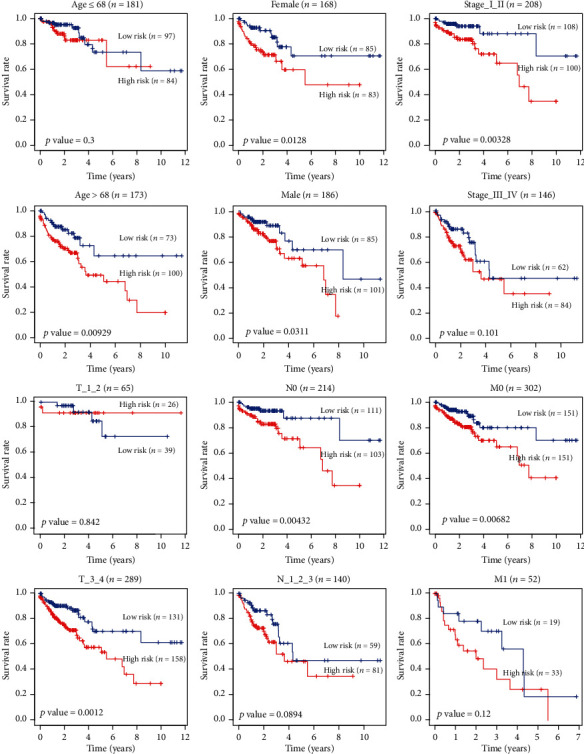
The OS of high- and low-risk groups in different clinical characteristics.

The area under ROC curve (AUC) of the receiver operating characteristic (ROC) curve was 0.694, suggesting that its predictive effectiveness of the OS was moderate (Figure 4(e)). In particular, it was worth mentioning that the AUC of our risk signature was larger than the existing clinic parameters stage, *T*, *N*, and *M*, which vigorously filled the blanks of existing clinical prognosis. To investigate our risk signature's independence, we performed univariate (Figure 4(f)) and multivariate Cox regression analysis (Figure 4(g)) and found that protein risk signature was an independent and reliable prognostic factor. The clinical characteristics of TCGA patients were shown in Table 1. 36 cases with incomplete clinical information were eliminated, and the remaining 354 CRC patients were finally evaluated. The median age of 354 CRC patients was 68 years. The OS was compared between high- and low-risk groups of different clinical characteristics (Figure 5).

### 3.4. Clinical Relevance Assessment

**Figure 6:**
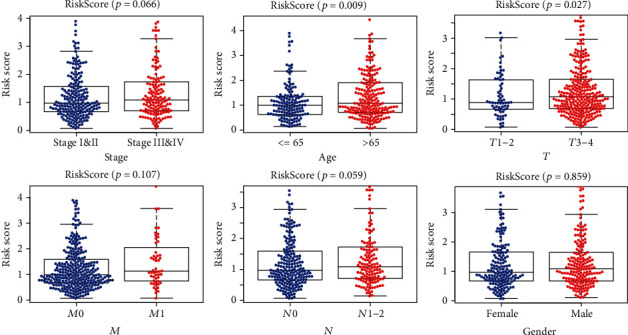
Relationships between protein risk signature and clinical characteristics. The risk score was significantly higher in T_3_4 than T_1_2, and the greater the age, the higher the risk score (*p* < 0.05). In terms of stage and *N*, the risk score had only a little short of significance (*p* > 0.05).

**Figure 7:**
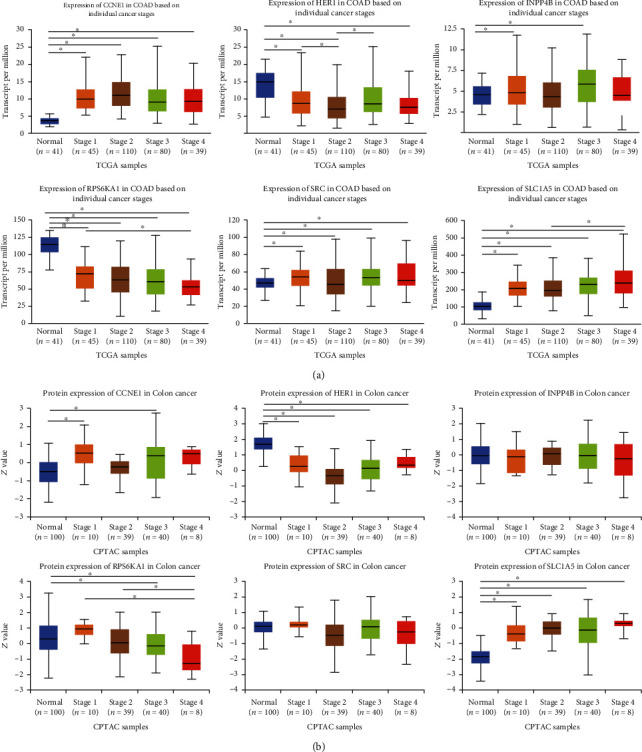
Relationships between expressions of 6 encoding genes and hub proteins and individual cancer stages of CRC patients (UALCAN). (a) mRNA expressions of HER1, RPS6KA1, and SLC1A5 were significantly associated with CRC patients' individual cancer stages. Patients who were in advanced stages tended to express higher HER1 and SLC1A5 and lower RPS6KA1. (b) At the protein expression level, the expression of the RPS6KA1 protein was lower in the advanced stage. ^∗^*p* < 0.05.

To understand our protein signature's clinical relevance, we investigated the relationships between the risk signature and age, gender, stage, *T*, *N*, and *M*. The risk score was significantly higher in seniors and advanced *T* stage cases (Figure 6). As shown in Figure 7(a), expressions of 6 encoding genes were significantly related to CRC patients' pathological stage. In the late stage, expressions of CCNE1, INPP4B, SRC, and SLC1A5 were significantly increased to varying degrees while expressions of HER1 and RPS6KA1 were significantly reduced to varying degrees. The same conclusion was reached at the protein level (Figure 7(b)).

### 3.5. Differential Expression Analysis of 6 Hub Proteins

**Figure 8:**
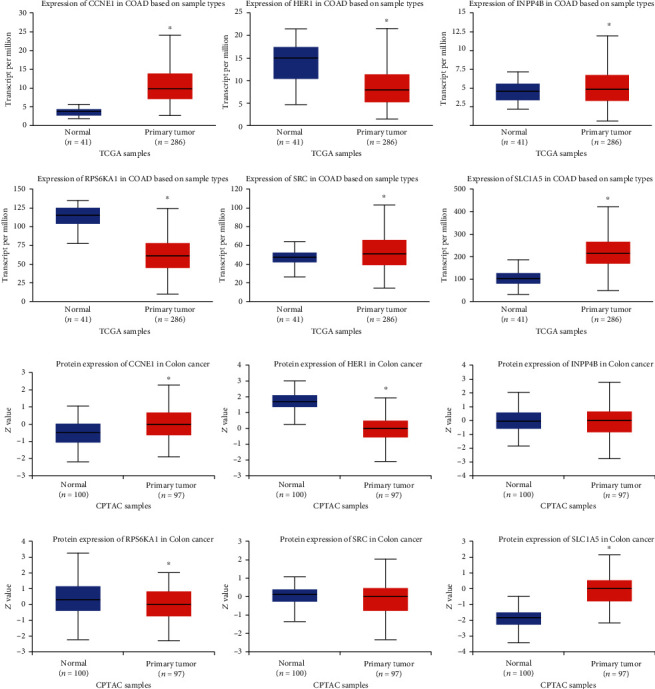
Expressions of 6 encoding genes and hub proteins between CRC tissues and normal tissues (UALCAN). CCNE1, INPP4B, SRC, and SLC1A5 were significantly overexpressed in CRC tissues while HER1 and RPS6KA1 were significantly underexpressed in CRC tissues. The mRNA expressions of INPP4B and SRC did not match their protein expression. ^∗^*p* < 0.05.

**Figure 9:**
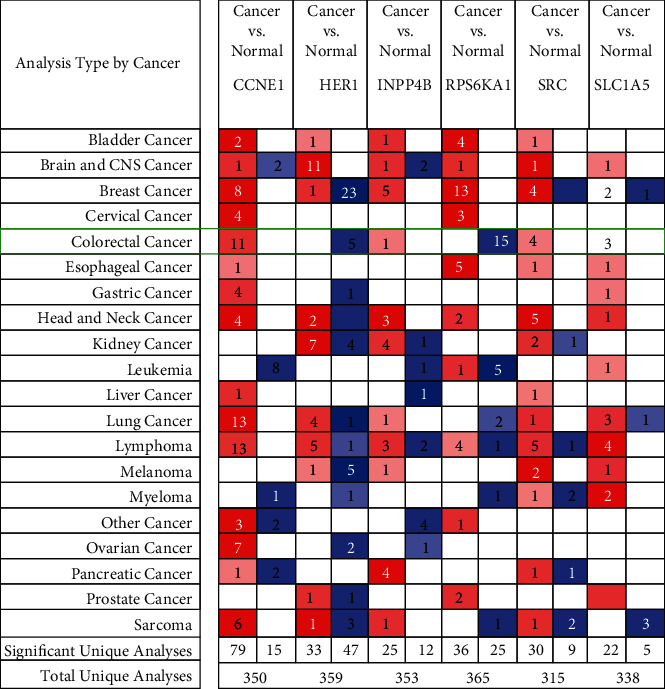
Transcriptional expressions of 6 encoding genes in 20 types of cancers at the ONCOMINE database. The difference in the mRNA expression was compared by Student's *t*-test. Red represented the overexpression while blue represented the low expression. The darker the color, the greater the significance of the over- or underexpressed genes.

Compared with normal samples, mRNA expressions of CCNE1, INPP4B, SRC, and SLC1A5 increased significantly in primary tumors. At the protein expression levels, we found that expressions of CCNE1 and SLC1A5 increased significantly in CRC tissues. Expressions of HER1 and RPS6KA1 in CRC were significantly reduced at both mRNA and protein levels (Figure 8). Next, we validated mRNA expressions of 6 encoding genes at the OMCOMINE website and finally drew the same conclusion (Figure 9).

### 3.6. Pearson Correlation Analysis of the TCPA Database

**Figure 10:**
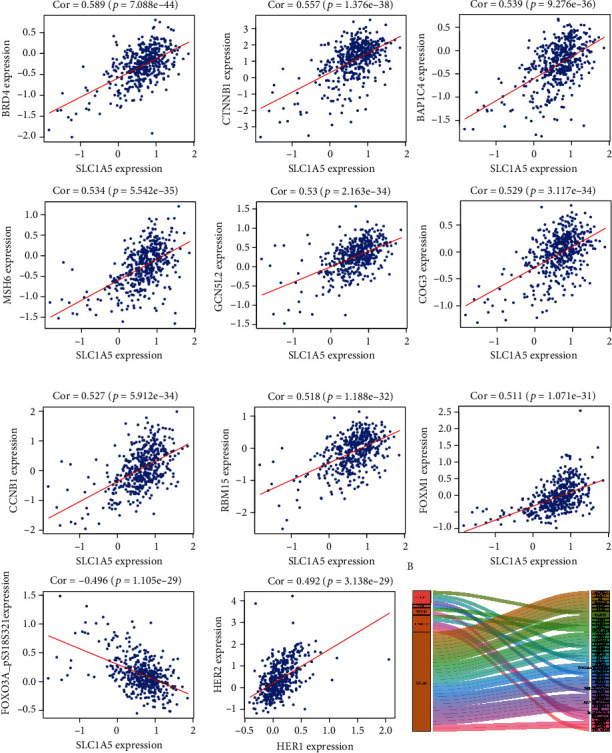
Coexpressed proteins of 6 hub proteins at the TCPA database. (a) 11 coexpressed proteins are moderately related to 6 hub proteins. Pearson correlation coefficient (PCC) was close to 0.5. (b) Sankey diagram of all proteins related to 6 hub proteins at the TCPA database (PCC > 0.4) (*p* < 0.05).

In organisms, proteins that have the same function or exist in the same pathway tend to be coexpressed. Based on the TCPA protein expression profile and 6 hub proteins, we extracted 11 significant coexpressed proteins (pearson correlation coefficient (PCC) was approximately equal to 0.5) (Figure 10(a)) and 29 coexpression proteins (PCC > 0.4). All of them were displayed in the Sankey diagram (Figure 10(b)).

### 3.7. Molecular Network and Functional Enrichment Analysis

**Figure 11:**
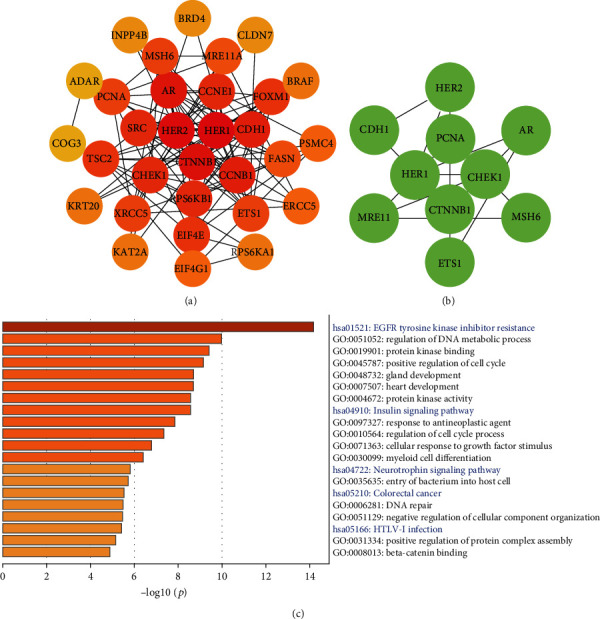
Protein-protein interaction (PPI) and enrichment analysis among coexpressed 46 proteins. (a) The PPI was ranked by degrees calculated by Cytoscape plugin cytoHubba. The darker the color, the higher the degree. (b) The significant module MOCDE_1 was built using the MCODE plugin at the Metascape. (c) The pathway and process enrichment analysis among 46 proteins. Blue words represented KEGG analysis, while black words represented GO analysis (*p* < 0.05).

Based on these 46 proteins, we performed protein-protein interaction (PPI) and functional enrichment analysis. PPI network demonstrated that HER1, HER2, and CTNNB1 were the top 3 nodes ranked by degrees calculated by Cytoscape plugin cytoHubba (Figure 11(a)). The Molecular Complex Detection (MCODE) algorithm had been applied to identify the densely connected network components, and the MCODE_1 was displayed in Figure 11(b). At the Metascape, we performed PPI enrichment analysis, and the top 3 clusters were displayed in Table 2. Nodes of MCODE_1 were mainly enriched in pathways in cancer (Table 2). Gene enrichment analysis mainly focused on EGFR (HER1) tyrosine kinase inhibitor resistance and regulation of the DNA metabolic process (Figure 11(c)). Enrichment analysis results at the protein, and gene levels all indicated that the prognostic-related proteins of CRC were related to EGFR tyrosine kinase inhibitor resistance.

## 4. Discussion

Incidence and mortality of colorectal cancer (CRC) in the United States rank third and second, respectively. The latest report states that CRC is getting younger and younger, and mortality increases with the delay of discovery. Advances in sequencing and microarray technology and the opening of various online databases have promoted the diagnosis and treatment of diseases at the genomic level.

Reviewing the previous literature on the prognosis of CRC, 4 gene signatures [28], 9 gene signatures [7], hypoxia-related signature [29], autophagy score signature [30, 31], somatic mutation signatures [32], metabolism-related signature [33], chemokine/chemokine receptor signature [34], and immune-related signature [35] had been constructed to predict the OS of CRC patients. However, these bioinformatic analyses only predict the prognosis of CRC patients at the RNA level, and there are few studies on the protein prognostic signature. Besides, these previous RNA bioinformatic studies did not identify the same protein-coding genes of our signature.

In this study, based on the TCPA and TCGA database, we performed K-M and Cox regression analysis. 6 hub proteins were screened, all of which were significantly associated with the overall survival (OS) of CRC patients. Based on these 6 hub proteins, we first developed a protein-related prognostic signature, which was an independent prognostic factor and significantly associated with CRC patients' OS. The receiver operating characteristic (ROC) curve and the areas under the ROC curve (AUC) further validated its accuracy. And our protein risk signature's performance was better than the existing clinical-pathological parameters, including *T*, *N*, *M*, and stage. Based on the median of the risk score, we divided CRC patients into high- and low-risk groups and found that CRC patients in the high-risk group had significantly more deaths than the low-risk group. Differential expression analysis found that 6 hub proteins were significantly differentially expressed in CRC patients with different stages and cancer tissues than normal tissues. Given the potential clinical significance of 6 hub proteins, we performed protein-protein interaction (PPI) and enrichment analysis on 6 hub proteins and their coexpressed proteins. The relationship between EGFR tyrosine kinase inhibitor resistance and CRC's development and prognosis urgently needs more research.

The CCNE1 protein (Cyclin E1) belongs to the highly conserved cyclin family, which forms a complex with CDK2 and functions as its regulatory subunit, whose activity is necessary for the G1/S transition in the cell cycle [36]. Previous studies have shown that CCNE1 amplification and overexpression result in chromosome instability and further lead to tumorigenesis, which is associated with poor prognosis in CRC [37].

Epidermal growth factor receptor (EGFR, HER1), belonging to the protein kinase superfamily, is a transmembrane glycoprotein that binds to epidermal growth factor, which induces receptor dimerization and tyrosine autophosphorylation and leads to cell proliferation [38, 39]. Another mechanism for the HER1 activation is the overproduction of its ligands. Previous studies have revealed that targeting HER1 can inhibit the proliferation and induce apoptosis of CRC cells [40]. Adding HER1 mAb to chemotherapy or best supportive care improves progression-free survival (moderate to high-quality evidence), the OS (moderate evidence), and tumor response rate (moderate to high-quality evidence) in CRC patients [41].

The inositol polyphosphate-4-phosphatase type IIB (INPP4B) protein belongs to the enzyme of the phosphatidylinositol signaling pathway. The mechanism of INPP4B is to remove the phosphate group of the inositol ring from inositol 3,4-bisphosphate. Previous studies have shown that INPP4B restrains CRC's proliferation and metastasis [42, 43]. However, some studies also demonstrated that NPP4B promotes survival and proliferation of tumor cells, including triple-negative breast cancer and leukemia [44–46].

Ribosomal protein S6 kinase A1 (RPS6KA1) is a member of the RSK (ribosomal S6 kinase) family of serine/threonine kinases and can phosphorylate members of the mitogen-activated kinase (MAPK) signaling pathway. RPS6KA1 participates in the carcinogenic process by regulating cell growth, insulin, and inflammation. Research also has shown that genetic variation of RPS6KA1 is significantly associated with the risk of developing colon cancer [47].

SRC proto-oncogene, nonreceptor tyrosine kinase (SRC), is the protein encoded by the proto-oncogene. Studies have shown that abnormal activation of intracellular tyrosine kinase SRC has been considered as a mechanism for acquired chemotherapy resistance in metastatic CRC [48]. SRC phosphorylation and activation can promote CRC invasion and metastasis [49, 50]. Stabilization of SRC promotes epithelial-mesenchymal transition in CRC [51]. And mutations of SRC can be involved in the malignant progression of colon cancer [52].

The solute carrier family 1 member 5 (SLC1A5) protein is a sodium-dependent neutral amino acid transporter, functioning as a receptor for RD114/type D retrovirus [53, 54]. Studies have found that SLC1A5 is an important transporter of glutamine, and upregulated SLC1A5 promotes the growth and survival of CRC cells [55]. And inhibition of SLC1A5 sensitizes CRC to cetuximab both in vitro and in vivo. The sensitization mechanism is that inhibition of SLC1A5 promotes ubiquitin-proteasome degradation of HER1 and reduces the nuclear HER1 expression [56].

Based on previous studies, these 6 proteins are involved in the development and resistance of CRC. Our study further reveals the prognostic roles of these 6 proteins in CRC. More molecular research is urgently needed.

In this study, at the Metascape, enrichment analysis revealed that 6 hub proteins and their coexpressed proteins mainly enriched in EGFR (HER1) tyrosine kinase inhibitor resistance. HER1 serves as a stimulus for cancer growth, and some tyrosine kinase inhibitor (TKI) targeting HER1 has been currently administered. However, TKI resistance is common and leads to the recurrence of tumors. The efficacy of combined targeting 6 hub proteins in patients with CRC needs to be further studied.

In summary, our studies revealed that differentially expressed 6 hub proteins and the protein risk signature were significantly associated with the OS of CRC patients. Our risk signature has the potential for clinical application to predict the outcomes of CRC patients, and 6 hub proteins are expected to become novel therapeutic targets in the future.

## Figures and Tables

**Table 1:** Clinical characteristics of 354 CRC patients included in our study.

Clinical characteristics	No. of patients (%)
Age	
≤68	181 (51.13)
>68	173 (48.87)
Gender	
Male	186 (52.54)
Female	168 (47.46)
Stage-AJCC	
Stage_I_II	208 (58.76)
Stage_III_IV	146 (41.24)
*T*	
T_1_2	65 (18.36)
T_3_4	289 (81.64)
*N*	
N0	214 (60.45)
N_1_2_3	140 (39.55)
*M*	
M0	302 (85.31)
M1	52 (14.69)

**Table 2:** PPI enrichment analysis at the Metascape.

GO	Description	Log10 (*p*)
hsa01521	EGFR tyrosine kinase inhibitor resistance	-15.2
hsa04012	ErbB signaling pathway	-10.8
hsa01522	Endocrine resistance	-10.4
MCODE_1_GO	Description	Log10 (*p*)
hsa05200	Pathways in cancer	-10.5
hsa05213	Endometrial cancer	-8.5
GO : 0048732	Gland development	-8.1

## Data Availability

The data used to support the findings of our study are available from the corresponding author upon request.

## Abbreviations

CRC: Colorectal cancer
TCPA: The Cancer Proteome Atlas
TCGA: The Cancer Genome Atlas
PPI: Protein-protein interaction
ROC curve: Receiver operating characteristic curve
AUC: Areas under the ROC curve
KEGG: Kyoto Encyclopedia of Genes and Genomes
GO: Gene ontology
OS: Overall survival
CCNE1: Cyclin E1
EGFR (HER1): Epidermal growth factor receptor
INPP4B: Inositol polyphosphate-4-phosphatase type IIB
RPS6KA1: Ribosomal protein S6 kinase A1
SRC: SRC proto-oncogene, nonreceptor tyrosine kinase
SLC1A5: Solute carrier family 1 member 5.
